# Brg1 Loss Attenuates Aberrant Wnt-Signalling and Prevents Wnt-Dependent Tumourigenesis in the Murine Small Intestine

**DOI:** 10.1371/journal.pgen.1004453

**Published:** 2014-07-10

**Authors:** Aliaksei Z. Holik, Madeleine Young, Joanna Krzystyniak, Geraint T. Williams, Daniel Metzger, Boris Y. Shorning, Alan R. Clarke

**Affiliations:** 1 Cardiff School of Biosciences, Cardiff University, Cardiff, United Kingdom; 2 Stem Cells and Cancer Division, The Walter and Eliza Hall Institute of Medical Research, Melbourne, Australia; 3 School of Medicine, Cardiff University, Cardiff, United Kingdom; 4 IGBMC, CNRS UMR7104/INSERM U964/Université de Strasbourg, Illkirch, France; National Cancer Institute, United States of America

## Abstract

Tumourigenesis within the intestine is potently driven by deregulation of the Wnt pathway, a process epigenetically regulated by the chromatin remodelling factor Brg1. We aimed to investigate this interdependency in an *in vivo* setting and assess the viability of Brg1 as a potential therapeutic target. Using a range of transgenic approaches, we deleted *Brg1* in the context of Wnt-activated murine small intestinal epithelium. Pan-epithelial loss of Brg1 using *VillinCreER^T2^* and *AhCreER^T^* transgenes attenuated expression of Wnt target genes, including a subset of stem cell-specific genes and suppressed Wnt-driven tumourigenesis improving animal survival. A similar increase in survival was observed when Wnt activation and Brg1 loss were restricted to the Lgr5 expressing intestinal stem cell population. We propose a mechanism whereby Brg1 function is required for aberrant Wnt signalling and ultimately for the maintenance of the tumour initiating cell compartment, such that loss of Brg1 in an Apc-deficient context suppresses adenoma formation. Our results highlight potential therapeutic value of targeting Brg1 and serve as a proof of concept that targeting the cells of origin of cancer may be of therapeutic relevance.

## Author Summary

Aberrant Wnt signalling is responsible for the majority of colorectal cancers, the third leading cause of cancer-related mortality in the UK. However, no therapies directly targeting Wnt signalling are currently available. Using mouse models of intestinal cancer, we demonstrate that deleting chromatin remodelling factor Brg1 in the context of Apc-deficient small intestinal epithelium attenuates Wnt-driven gene expression changes and prevents adenoma formation, which results in extended animal survival. We also demonstrate that Brg1 loss impairs the small intestinal stem cell expansion associated with aberrant activation of Wnt signalling. These findings highlight Brg1 as a potential therapeutic target in Wnt-driven intestinal tumourigenesis and illustrate the viability of targeting the somatic stem cell as the ‘cell of origin’ of cancer, which might be particularly valuable in patients with known predisposition to cancer.

## Introduction

More than 90% of colorectal cancers (CRC) are characterised by aberrant activation of the canonical Wnt/β-catenin pathway, which is proposed to play a major role in the initiation and progression of CRC [1]. Despite this clear link between deregulated Wnt signalling and disease, therapies which target the Wnt pathway remain limited [2]. There is, therefore, a substantial demand for novel approaches to inhibit the Wnt pathway, preferably downstream of common aberrations such as mutations in *APC*, *AXIN2* or *β-catenin*. The emerging plethora of epigenetic factors involved in DNA methylation, histone modifications and chromatin remodelling represent a set of new and relatively unexplored opportunities for such therapeutic intervention [3].

*Brahma-related gene 1* (*BRG1*) or *SWI/SNF-related matrix-associated actin- dependent regulator of chromatin subfamily A member 4* (*SMARCA4*) is one of the two mutually exclusive ATPase subunits of the 2-MD family of SWItch/Sucrose Non-Fermentable (SWI/SNF) class of chromatin remodelling complexes. BRG1 has been implicated in a variety of biological processes, in both normal and neoplastic tissues [4], [5]. The majority of these studies, both *in vitro* and *in vivo*, suggest that BRG1 acts as a tumour suppressor. For example, it has been found to be mutated in numerous cancer cell lines and primary cancers [6]–[9]. In support of this, studies using Brg1 knock-out mouse models have shown that heterozygous loss of Brg1 increases susceptibility to both mammary gland and lung tumourigenesis [10], [11].

By contrast, BRG1 has been shown to interact with β-catenin and facilitate trans-activation of Wnt-dependent reporter assays and endogenous Wnt target genes in cancer cell lines [12], [13]. Therefore better understanding of the role of BRG1 in aberrant Wnt signalling may allow the development of novel Wnt intervention strategies.

We have recently reported that loss of Brg1 in the small intestinal epithelium results in depletion of the intestinal stem cell population [14]. In this study we aimed to investigate the functional interaction between Brg1 and the Wnt pathway by generating mice, which carried floxed alleles of both *Apc* [15] and *Brg1* [16], [17] genes, thus placing Brg1 deficiency in the context of aberrant activation of the Wnt pathway.

Using three different conditional approaches, we find that additional loss of Brg1 from the small intestinal epithelium attenuates the Wnt-dependent phenotype resulting from Apc deletion.

## Results

### Brg1 loss attenuates aberrant Wnt signalling in the murine small intestinal epithelium

In order to assess the immediate consequences of Brg1 loss upon Wnt activation, we employed the (*Tg(Vil-cre/ESR1)23Syr*) transgene [18] driving expression of Cre-ER^T2^ recombinase under control of the *Villin 1* promoter, further abbreviated as *VillinCreER^T2^*. To achieve high penetrance inactivation of targeted genes, *VillinCreER^T2+^Apc^fl/fl^* and *VillinCreER^T2+^Apc^fl/fl^Brg^fl/fl^* mice along with *VillinCreER^T2−^* controls were induced with four daily injections of 80 mg/kg Tamoxifen. We sacrificed the mice 4 days after the first induction and collected samples of the small intestinal epithelium.

**Figure 1:**
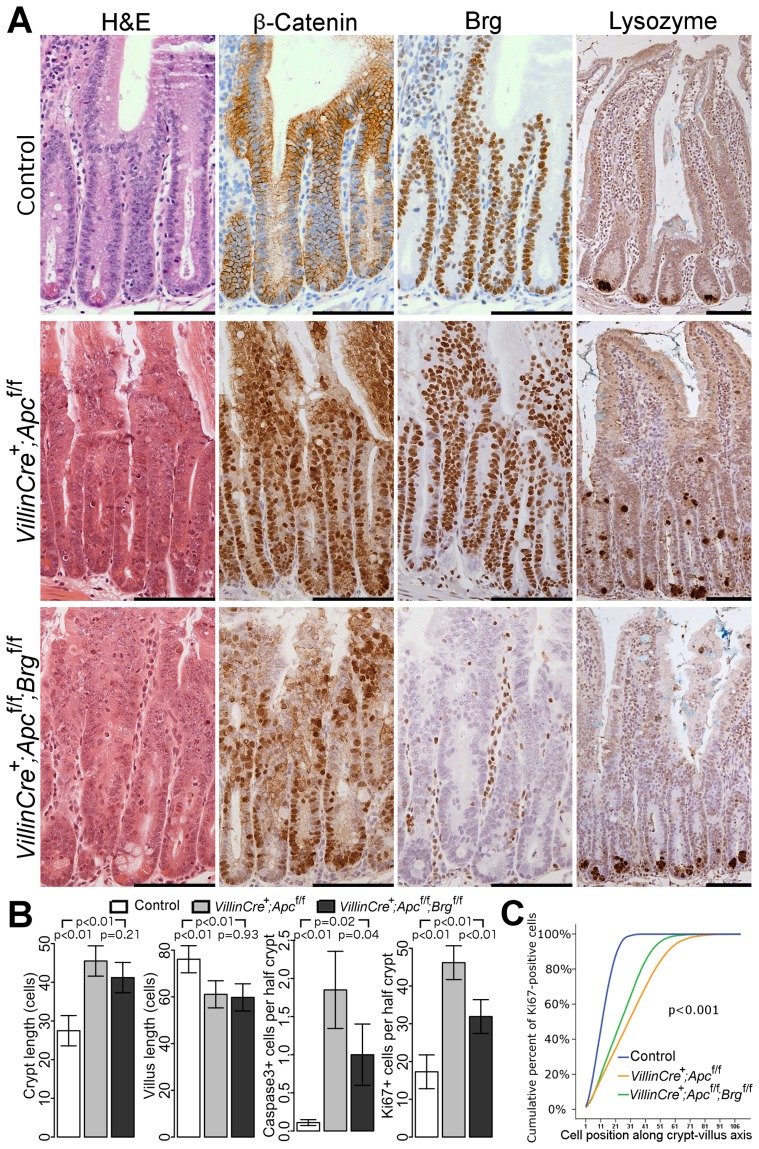
Brg1 loss attenuates the effects of Apc deletion in the small intestinal epithelium. A. H&E, β-catenin and Brg1 staining of the small intestinal epithelium from control *VillinCreER^T2−^*, *VillinCreER^T2+^Apc^fl/fl^* and *VillinCreER^T2+^Apc^fl/fl^Brg^fl/fl^* mice 4 days after high-dose induction revealed disturbed crypt architecture and nuclear localisation of β-catenin in both cohorts, as well as complete loss of Brg1 in *VillinCreER^T2+^Apc^fl/fl^Brg^fl/fl^* epithelium. Lysozyme immunostaining showed mis-localisation of Paneth cells in *VillinCreER^T2+^Apc^fl/fl^* epithelium, which was restored to normal by inactivation of Brg1. Scale bars represent 100 µm. B. Scoring of the crypt and villus length revealed no differences between double knock-out mice (black) and *VillinCreER^T2+^Apc^fl/fl^* animals (grey). Scoring of the cleaved Caspase3 positive cells and Ki67 positive cells detected a decrease in both apoptosis and proliferation in *VillinCreER^T2+^Apc^fl/fl^Brg^fl/fl^* mice compared to *VillinCreER^T2+^Apc^fl/fl^* animals. Data are shown as mean ± group's standard deviation for Caspase3 quantification and as mean ± pooled standard deviation otherwise, p value was calculated by means of t test not assuming equal variance for Caspase3 data and by means of one way ANOVA otherwise, p value was adjusted for multiple testing. For all comparisons n≥4. C. Analysis of cumulative frequency of Ki67 positive cells at each position along crypt-villus axis revealed expansion of the proliferative compartment in both double knock-out (green line) and *VillinCreER^T2+^Apc^fl/fl^* (orange line) mice compared to *VillinCreER^T2−^* controls (blue line). This expansion was less pronounced in double knock-out mice compared to *VillinCreER^T2+^Apc^fl/fl^* animals. For all pair wise comparisons Kolmogorov-Smirnov test p<0.001, n = 4.

Immunohistochemical analysis of β-catenin and Brg1 expression in the jejunum epithelium at day 4 post induction revealed nuclear localisation of β-catenin in both *VillinCreER^T2+^Apc^fl/fl^* and *VillinCreER^T2+^Apc^fl/fl^Brg^fl/fl^* mice as well as complete loss of Brg1 in double knock-out animals (Figure 1A). Upon histological inspection, the small intestinal epithelium of both *VillinCreER^T2+^Apc^fl/fl^* and *VillinCreER^T2+^Apc^fl/fl^Brg^fl/fl^* mice displayed an aberrant proliferative and apoptotic response synonymous with Wnt pathway activation [19] (Figure 1A). Although no difference in crypt and villus length was observed between *VillinCreER^T2+^Apc^fl/fl^* and *VillinCreER^T2+^Apc^fl/fl^Brg^fl/fl^* mice (Figure 1B, p>0.05, n≥4), quantitative analysis of other histological parameters such as apoptosis and mitosis levels as well as 5-bromo-2′-deoxyuridine (BrdU) incorporation 2 hours after a BrdU pulse showed significant differences between the experimental groups (Figure 1B–1C, Figure S1A–S1B). Quantification of cleaved Caspase3 positive cells showed reduced apoptosis in *VillinCreER^T2+^Apc^fl/fl^Brg^fl/fl^* epithelium (1.85±0.51 and 1.0±0.40 Caspase3 positive cells per half-crypt, p = 0.04, n = 4, Figure 1B). This observation was supported by scoring of the number of apoptotic bodies in the jejunum of *VillinCreER^T2+^Apc^fl/fl^* and *VillinCreER^T2+^Apc^fl/fl^Brg^fl/fl^* mice (4.09±0.34 and 2.67±0.45, p<0.01, n≥4, Figure S1A). Quantification of Ki67 positive cells revealed significantly reduced proliferation in the small intestine of double knock-out mice compared to their Apc-deficient counterparts (46.18 and 31.91 positive cells per half-crypt, pooled standard deviation 4.54, p = 0.0004, n≥4, Figure 1B). Consistent with this observation, scoring of BrdU positive cells 2 hours after labelling showed reduced BrdU incorporation in the jejunum of *VillinCreER^T2+^Apc^fl/fl^Brg^fl/fl^* mice (32.51±6.38 and 23.91±0.99, p = 0.038, n≥4, Figure S1B). Cumulative distribution analysis of both Ki67 and BrdU positive cells revealed that the expansion of the proliferative compartment resulting from Apc deletion was attenuated by additional loss of Brg1 (Figure 1C and Figure S1C, for all comparisons Kolmogorov-Smirnov p<0.001).

**Figure 2:**
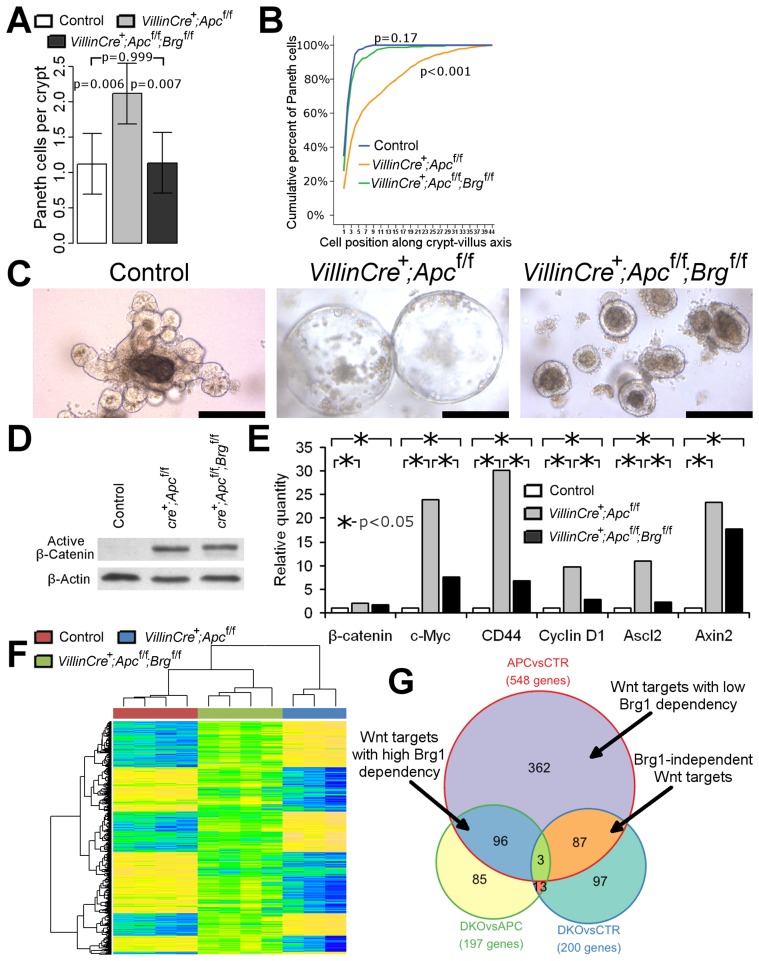
Brg1 loss attenuates Wnt target gene expression and prevents mislocalisation of Paneth cells and formation of aberrant organoids. A. Scoring of the lysozyme positive cells revealed a significant increase in number of Paneth cells in *VillinCreER^T2+^Apc^fl/fl^* (grey bar) mice compared to double knock-out (black bar) and control (white bar) mice. No difference in Paneth cell numbers was observed between double knock-out and control epithelium. Data are shown as mean ± pooled standard deviation, p value was calculated by means of one way ANOVA, n = 4. B. Analysis of cumulative frequency of Paneth cells at each position along crypt-villus axis revealed their expansion into the upper portion of the crypt in *VillinCreER^T2+^Apc^fl/fl^* epithelium compared to double knock-out and control mice (Kolmogorov-Smirnov test p<0.001, n = 4). No difference in Paneth cell distribution was observed between double knock-out and control epithelium (Kolmogorov-Smirnov test p = 0.17, n = 4). C. Organoid culture of crypts derived from *VillinCreER^T2+^Apc^fl/fl^* epithelium produced large cystic organoids by day 6 of culture (day 9 post induction). Meanwhile, very few large cysts developed from *VillinCreER^T2+^Apc^fl/fl^Brg^fl/fl^* crypts, with most crypts producing compact simple organoids. Crypts from control intestinal epithelium developed into complex organoid structures by the same time point. Scale bars represent 200 µm. D. Western-blotting analysis of total protein pooled from 3 different animals for each genotype did not reveal changes in the levels of activated β-catenin between *VillinCreER^T2+^Apc^fl/fl^* and *VillinCreER^T2+^Apc^fl/fl^Brg^fl/fl^* epithelial samples. Substantially weaker signal was detected in the samples from *VillinCreER^T2−^* control animals. E. qRT-PCR analysis of total RNA extracted from the small intestinal epithelium revealed significantly increased expression of *β-catenin* and a range of Wnt target genes in *VillinCreER^T2+^Apc^fl/fl^* (grey bars) and *VillinCreER^T2+^Apc^fl/fl^Brg^fl/fl^* (black bars) samples compared to control *VillinCreER^T2−^* (white bars) animals. Expression levels of *c-Myc*, *CD44*, *CyclinD1* and *Ascl2*, but not *β-catenin* or *Axin2* genes were found to be significantly lower in double knock-out epithelium compared to *VillinCreER^T2+^Apc^fl/fl^* samples. Mann-Whitney U-test was performed on ΔΔCt values, * - p<0.05, n≥4. F. Clustering analysis of genes differentially expressed between *VillinCreER^T2+^Apc^fl/fl^* and *VillinCreER^T2−^* control epithelium revealed that the same genes in *VillinCreER^T2+^Apc^fl/fl^Brg^fl/fl^* epithelium largely assumed intermediate expression values between those of the other two groups. G. Venn diagram demonstrating overlap between pairwise comparisons of differentially expressed gene signatures within various cohorts.

Interestingly, immunostaining with lysozyme antibody showed a difference in the distribution of Paneth cells between *VillinCreER^T2+^Apc^fl/fl^Brg^fl/fl^* and *VillinCreER^T2+^Apc^fl/fl^* mice. Small intestinal epithelium of *VillinCreER^T2+^Apc^fl/fl^* animals displayed mislocalisation of Paneth cells throughout the crypt (Figure 1A, right panels), consistent with our earlier report of Apc loss [19]. Additional deletion of Brg1 in *VillinCreER^T2+^Apc^fl/fl^Brg^fl/fl^* mice restored normal confinement of Paneth cells to the crypt base (Figure 1A, right panels). Quantitative analysis of Paneth cell number and position confirmed this observation as both number and distribution of Paneth cells in *VillinCreER^T2+^Apc^fl/fl^Brg^fl/fl^* epithelium were indistinguishable from normal mucosa (Figure 2A–2B, for Paneth cell number p = 0.999, for Paneth cell position Kolmogorov-Smirnov p = 0.17).

Unfortunately, high dose induction resulted in rapid health deterioration of both *VillinCreER^T2+^Apc^fl/fl^Brg^fl/fl^* and *VillinCreER^T2+^Apc^fl/fl^* genotypes such that animals had to be killed by 4 days post induction. We therefore used the *in vitro* intestinal organoid system to investigate the fate of double knock-out small intestinal crypts beyond this time point. *VillinCreER^T2+^Apc^fl/fl^* and *VillinCreER^T2+^Apc^fl/fl^Brg^fl/fl^* mice along with *VillinCreER^T2−^* controls were induced by 3 daily intraperitoneal injections of 3 mg Tamoxifen and sacrificed at day 3 post induction. Small intestinal crypts were isolated and placed in crypt organoid media lacking R-spondin (crypts from control animals were grown in presence of R-spondin). By day 6 of culture (day 9 post induction) small intestinal crypts from control animals became complex organoids (Figure 2C). At the same time, the majority of *VillinCreER^T2+^Apc^fl/fl^* crypts developed into large cystic structures (Figure 2C) consistent with a previous report of organoid culture under Wnt activated conditions [20]. In contrast, crypts derived from *VillinCreER^T2+^Apc^fl/fl^Brg^fl/fl^* epithelium rarely developed into large cysts and instead the majority remained as small simple spherical organoids indicating that double knock-out crypts had limited proliferative capacity despite constitutive activation of Wnt signalling (Figure 2C).

Brg1 loss therefore attenuated the histological and physiological consequences of Apc loss, implicating a compromised Wnt pathway activity. Notably, we consistently observed fewer cells with nuclear β-catenin in the epithelium of double knock-out mice compared to the *VillinCreER^T2+^Apc^fl/fl^* animals. Since nuclear β-catenin is commonly used as a surrogate marker of β-catenin activation, we wished to investigate if Brg1 loss suppressed Wnt signalling upstream of β-catenin activation. Western-blotting analysis of ‘activated’ β-catenin (dephosphorylated on Ser37 or Thr41) levels in the intestinal epithelium at day 4 after high-dose induction did not reveal any differences between the two experimental groups (Figure 2D), suggesting that Brg1 deficiency was likely to suppress the Wnt pathway downstream of β-catenin activation.

In order to further investigate the consequences of Brg1 loss on the Wnt pathway, we assessed expression levels of known Wnt target genes in the small intestinal epithelium of *VillinCreER^T2+^Apc^fl/fl^* and double knock-out mice. qRT-PCR analysis of *CD44*, *c-Myc*, *CyclinD1* and *Ascl2* expression levels revealed significant down-regulation of these genes in *VillinCreER^T2+^Apc^fl/fl^Brg^fl/fl^* mice compared to *VillinCreER^T2+^Apc^fl/fl^* epithelium (Figure 2E, p<0.05, n≥3). Notably, we did not observe a significant difference in expression levels of Axin2, which is commonly used as a readout of Wnt pathway activation [21], indicating possible differential recruitment of Brg1 to distinct Wnt target genes.

To further investigate the small intestine-specific role of Brg1 in the Wnt mediated transcriptional program, we performed genome-wide expression analysis of Apc-deficient and double knock-out (DKO) epithelium. *VillinCreER^T2+^Apc^fl/fl^* (n = 3) and *VillinCreER^T2+^Apc^fl/fl^Brg^fl/fl^* (n = 4) mice along with *VillinCreER^T2−^* controls (n = 4) were induced with four daily injections of 80 mg/kg Tamoxifen and whole epithelial extract was harvested at day 4 post induction. RNA was extracted from epithelial samples, labelled and hybridised to Mouse Ref8 v2 Illumina array. To determine the baseline effect of Brg1 loss, we performed similar analyses on wild type and induced *VillinCreER^T2+^Brg^fl/fl^* samples, which are described in our earlier publication [14].

Analysis of gene expression between Wnt activated *VillinCreER^T2+^Apc^fl/fl^* and control epithelium (APCvsCTR gene set) detected 548 unique differentially expressed ‘Wnt target genes’ including a number of previously reported Wnt target genes, such as *Cd44*, *Axin2*, *Lgr5*, *Tiam1*, *Ephb2*, *Efna4* and *Sox9* [22] (Figure 2G, Table S1) with four of these genes found to be down-regulated in DKO epithelium compared to Apc-deficient samples (Table S2). Cluster analysis of genes differentially expressed between Apc-deficient and control epithelium revealed clustering of DKO mice with control samples (Figure 2F), indicating a greater similarity of the gene expression pattern between DKO and wild type samples compared to Apc-deficient intestine. Notably, the majority of gene expression values from DKO samples were intermediate between those of *VillinCreER^T2+^Apc^fl/fl^* and control samples (Figure 2F), indicating attenuation of the Wnt pathway transcriptional signature regardless of whether Wnt activation induced or suppressed gene expression. We also observed a strong negative correlation pattern when directions of gene expression changes between Apc-deficient and wild type epithelium were contrasted with changes between double knock-out and Apc-deficient mice (Figure S2A, ρ = −0.793, p<0.0001).

In order to evaluate how many Wnt target genes were attenuated by additional Brg1 loss we compared the overlap between the sets of differentially expressed genes (Figure 2G). We identified 197 unique differentially expressed genes between *VillinCreER^T2+^Apc^fl/fl^* and *VillinCreER^T2+^Apc^fl/fl^Brg^fl/fl^* samples (DKOvsAPC set) (Table S2). Of these, half (99/197) appeared to be ‘Wnt targets’ defined as those present in APCvsCTR gene set (Figure 2G, Table S1), representing 18% (99/548) of the total number of genes that were deregulated following Apc loss (Table S4, Figure 2G). Since Brg1 loss was able to significantly attenuate deregulation of these Wnt targets, these genes could be designated as ‘highly dependent on Brg1’ (Table S7). Comparison of gene expression between *VillinCreER^T2+^Apc^fl/fl^Brg^fl/fl^* and control samples (DKOvsCTR gene set) identified 200 unique differentially expressed genes (Figure 2G, Table S3). Comparison of this gene set to the APCvsCTR set revealed that 16% (87/548) of ‘Wnt target genes’ were present in both sets, but not in the DKOvsAPC set (Table S5). Since Brg1 loss failed to attenuate deregulation of these genes, they could be considered as ‘Brg1 independent Wnt targets’ (Table S7). Notably, Brg1 deficiency in double knock-out samples prevented deregulation of 84% (458/548) of Wnt target genes from their expression levels in control intestine. With the exception of the 99 highly Brg1 dependent Wnt targets these 66% (362/548) of genes could be designated as ‘Wnt targets with low Brg1 dependency’ (Table S7). This gene subset included such Wnt targets as *Axin2*, *Ephb2* and *Sox9*. Overall, these data indicated that up to 84% of all Wnt target genes in the small intestinal epithelium are dependent to some extent on Brg1 for either their activation or suppression.

It could be argued that gene expression changes upon additional deletion of Brg1 in the Apc-deficient epithelium could arise from the global effect of Brg1 loss on gene expression rather than its specific relevance for the Wnt-driven transcriptional programme. To address this caveat we also analysed the genes differentially expressed between Brg1-deficient and wild type epithelium. Despite a drastic effect on epithelial homeostasis, Brg1 loss induced a relatively moderate perturbation in gene expression at day 4 post induction with 86 genes differentially expressed between Brg1-deficient and control epithelium (Table S8) [14]. Expectedly, a small proportion of the genes differentially expressed between Apc-deficient and double knock-out samples were also de-regulated between Brg1-deficient and wild type epithelium (23/197 genes (11.7%) Table S9, Figure S2B). This proportion was notably lower when Brg1 targets were compared to the list of Wnt targets with high Brg1 dependency (5/99 genes (5.1%), Table S9). Together, these observations strongly suggested that gene expression changes following Brg1 loss in the context of Apc deficiency were largely a result of a specific effect of Brg1 deficiency on the Wnt pathway transcriptional programme rather than an impact of Brg1 deletion on global gene expression.

16 genes which overlapped between the DKOvsCTR and DKOvsAPC sets (Table S6, Figure 2G) appeared deregulated by Brg1 deficiency regardless of Wnt activation and therefore most likely represented direct Brg1 targets independent of Wnt signalling. Most of these genes (11/16) were also present among genes differentially expressed between Brg1-deficient and control samples (Table S9, Figure S2C) [14].

Notably, one of the Wnt targets, whose expression was attenuated following additional loss of Brg1, was the proposed intestinal stem cell marker Lgr5 [23]. We therefore decided to explore the effects of Apc deletion and subsequent Brg1 loss on the expression of genes associated with the intestinal stem cell compartment. To this end we compared gene expression changes between our cohorts to the extensive stem cell signature. Using 3 distinct genome-wide expression platforms Muñoz *et al.* identified 510 genes that were preferentially expressed in the murine small intestinal Lgr5^high^ cells [24]. We identified 460 of these stem cell-specific genes in our array, which corresponded to 721 probes (Table S10).

We then employed ROAST function [25] from the limma Bioconductor package [26] to determine if stem cell-specific genes were enriched among genes differentially expressed between our cohorts. Consistent with the reported stem cell expansion following aberrant activation of Wnt signalling [27]–[29], 50.5% of stem cell-specific genes were found to be up-regulated in *VillinCreER^T2+^Apc^fl/fl^* mice compared to the control cohort (p<0.0001). Double knock-out epithelium also displayed increased expression of stem cell-related genes compared to control samples, however only in 32.3% of genes (p = 0.0002). In contrast, 41.2% of all stem cell genes were suppressed in double knock-out epithelium compared to *VillinCreER^T2+^Apc^fl/fl^* mice (p<0.0001).

We also queried sets of differentially expressed genes between our cohorts for the presence of stem cell-specific genes. While this approach was less sensitive than using ROAST function, we observed a substantial number of stem cell signature genes in our differentially expressed gene sets (Tables S11, S12, S13). In line with the proposed Wnt-driven stem cell expansion [27]–[29], 10.6% (58/548) of all genes differentially expressed upon Apc deletion were found to belong to the stem cell signature. Of these 98% (57/58) were found to be up-regulated. In contrast, genes differentially expressed between double knock-out and Apc-deficient intestine contained 11.2% (22/197) of genes from the stem cell signature, all of which were down-regulated (Tables S11, S12, S13). Similar to the pattern of gene expression changes across all genes, stem cell related genes displayed strong negative correlation, when expression changes between Apc-deficient and wild type epithelium were contrasted with ones between double knock-out and Wnt activated intestine (Figure S2D, ρ = −0.859, p<0.0001).

Notably, the proposed Wnt-independent intestinal stem cell marker *Olfm4* [27] was found to be down regulated in the double knock-out intestine when compared to both Apc-deficient and normal epithelium, indicating strong Brg1 control over *Olfm4* expression.

### Brg1 loss prevents Wnt-driven adenoma formation in the context of the murine small intestinal epithelium

The apparent requirement of Brg1 for the expression of Wnt target genes following aberrant Wnt activation in the small intestinal epithelium raises the possibility that Brg1 loss may also prevent Wnt-driven tumour development. To explore this possibility we assessed the effects of Brg1 loss on Wnt-driven adenoma formation by driving recombination of the floxed *Apc* and *Brg1* alleles with the *Tg(Cyp1a1-cre/ESR1)1Dwi* transgene, further abbreviated as *AhCreER^T^* [30]. This transgene encodes Cre-ER^T^ recombinase under the control of the *Cyp1A* promoter. In contrast to *VillinCreER^T2^* recombinase, which is expressed in the entirety of the intestinal epithelium, *AhCreER^T^* transgene's expression is confined to the stem cell and early progenitor compartments. Additionally, *AhCreER^T^* recombinase requires exposure to both β-naphthoflavone and tamoxifen for its activation, which results in tighter control over its activity. We used this approach to inactivate Apc and Brg1 in the small intestinal epithelium at a lower frequency than above, thus extending animal survival and enabling us to analyse the long-term effects of Brg1 loss on Wnt-driven adenoma formation.

We induced 3 cohorts of mice (*AhCreER^T−^* controls, *AhCreER^T+^Apc^fl/fl^* and *AhCreER^T+^Apc^fl/fl^Brg^fl/fl^*) with 5 bi-daily intraperitoneal injections of 80 mg/kg of β-naphthoflavone and 80 mg/kg of tamoxifen. Animals were aged and sacrificed either at day 10 post induction (n = 4) or when they displayed signs of terminal illness (n≥20).

**Figure 3:**
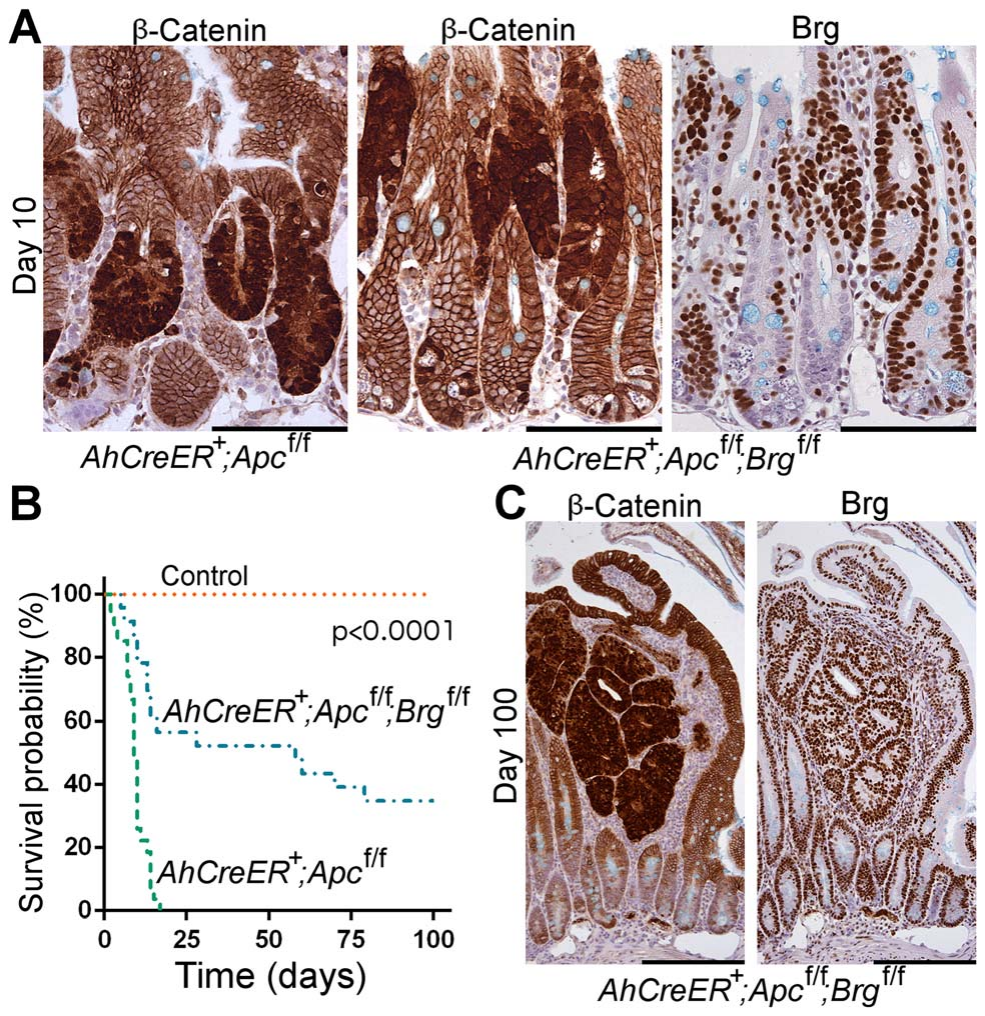
Brg1 loss is incompatible with Wnt-driven adenoma formation and improves animal survival upon Apc inactivation in the small intestine. A. Immunohistochemical analysis of β-catenin revealed numerous lesions with nuclear localisation of β-catenin in the small intestine of *AhCreER^T+^Apc^fl/fl^* and *AhCreER^T+^Apc^fl/fl^Brg^fl/fl^* mice 10 days post induction. Brg1 immunostaining of serial sections showed clusters of Brg1-deficient cells in *AhCreER^T+^Apc^fl/fl^Brg^fl/fl^* intestine, but failed to detect any overlap between lesions with nuclear β-catenin and Brg1 negative cells. B. Survival analysis demonstrated significantly increased survival probability of *AhCreER^T+^Apc^fl/fl^Brg^fl/fl^* mice (blue line, median survival 58 days) compared to *AhCreER^T+^Apc^fl/fl^* animals (green line, median survival 9 days). Log-rank test p<0.0001, n≥20. No control animals died within the timeframe of the experiment (n = 8). C. β-catenin immunostaining of the small intestinal epithelium of *AhCreER^T+^Apc^fl/fl^Brg^fl/fl^* mice 100 days post induction revealed numerous micro adenomas enclosed within distended villi. Staining with Brg1 antibody showed that all the micro adenomas retained Brg1 expression. Scale bars represent 100 µm (A) and 200 µm (B).

Immunohistochemical analysis of β-catenin localisation in the small intestinal epithelium at day 10 revealed multiple aberrant foci with nuclear β-catenin in both *AhCreER^T+^Apc^fl/fl^* and double knock-out mice, indicating successful activation of the Wnt pathway in those lesions (Figure 3A, left and central). At the same time, Brg1 immunostaining revealed clusters of Brg1 negative cells in *AhCreER^T+^Apc^fl/fl^Brg^fl/fl^* intestinal epithelium (Figure 3A, right). In contrast to the small intestine of *VillinCreER^T2+^Apc^fl/fl^Brg^fl/fl^* mice, where the majority of cells after induction were deficient for Brg1 and displayed nuclear localisation of β-catenin, all the lesions with nuclear β-catenin in the *AhCreER^T+^Apc^fl/fl^Brg^fl/fl^* intestine were Brg1-positive and we failed to detect any overlap between Brg1-deficient clusters and Wnt-activated lesions (Figure 3A, central and right). This observation suggested that, when driven by *AhCreER^T^* recombinase, Brg1 loss was incompatible with long term activation of the Wnt pathway and the development of aberrant crypt foci, consistent with inability of crypts from double knock-out epithelium to form aberrant organoids *in vitro* (Figure 2C).

Analysis of the ageing cohorts revealed that combined deletion of Apc and Brg1 deletion provided a survival advantage in comparison to single Apc deletion (Figure 3B). Whilst all the *AhCreER^T+^Apc^fl/fl^* mice became terminally ill within 20 days post induction (median survival 9 days), the majority of *AhCreER^T+^Apc^fl/fl^Brg^fl/fl^* mice survived substantially longer (median survival 58 days) with some mice surviving past 100 days (Figure 3B, Log-rank test p<0.0001, n≥20 for each cohort). No control animals (n = 8) developed signs of ill health within the timeframe of the experiment.

Histological inspection of the small intestine of the *AhCreER^T+^Apc^fl/fl^Brg^fl/fl^* mice at late time points revealed numerous small lesions confined within the distended villi and identified as micro-adenomas (Figure 3C), as well as rare large adenomas. Immunohistochemical analysis of β-catenin expression showed that all of these lesions were positive for nuclear β-catenin, confirming aberrant Wnt signalling activation (Figure 3C, left). Notably, Brg1 staining demonstrated that all the lesions retained Brg1 expression (Figure 3C, right), indicating that they were likely to originate from cells that recombined at the *Apc*, but not at the *Brg1* loci. Along with the apparent lack of double mutant lesions at day 10 post induction, this indicated that long-term progression of Wnt-driven neoplasia in the small intestine was incompatible with Brg1 deficiency. We therefore explored the possibility that Brg1 loss in the context of activated Wnt signalling could reduce tumour burden and thus improve animal survival. We induced *AhCreER^T+^Apc^fl/fl^* and *AhCreER^T+^Apc^fl/fl^Brg^fl/fl^* mice with two bi-daily injections of 80 mg/kg β-naphthoflavone and 80 mg/kg tamoxifen to achieve attenuated recombination and harvested the small intestinal epithelium at day 40 post induction. We then scored the number of lesions with nuclear β-catenin normalised to the number of normal crypt units contained within the analysed region of the small intestinal epithelium. Quantitative analysis of the tumour burden from two independent experiments revealed a 2.92-fold decrease in the number of lesions in the double knock-out small intestinal epithelium compared to *AhCreER^T+^Apc^fl/fl^* animals (p = 0.026, n≥5) indicating reduced tumour burden upon Brg1 loss.

### Stem cell-restricted Brg1 loss suppressed Wnt-driven tumourigenesis

**Figure 4:**
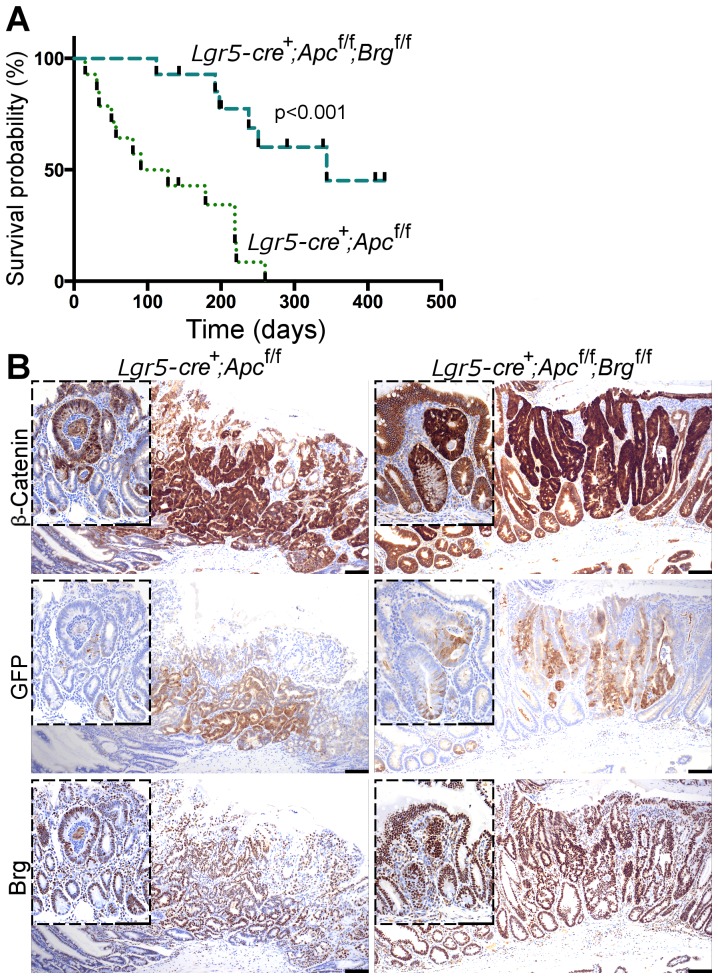
Stem cell-specific Brg1 loss in the small intestine attenuates Wnt-driven adenoma formation and improves animal survival. A. Survival analysis demonstrated significantly increased survival of *Lgr5-GFP-CreER^T2+^Apc^fl/fl^Brg^fl/fl^* mice (median survival 344 days post induction) compared to *Lgr5-GFP-CreER^T2+^Apc^fl/fl^* animals (median survival 110 days). Log-rank test p<0.0001, n = 14. B. β-catenin immunostaining detected micro and macro adenomas with nuclear β-catenin in the distal small intestinal epithelium of *Lgr5-GFP-CreER^T2+^Apc^fl/fl^* and *Lgr5-GFP-CreER^T2+^Apc^fl/fl^Brg^fl/fl^* mice at various time points. Both types of lesions were found to express GFP and Brg1 indicating their stem cell origin and selective elimination of Brg1-deficient tumours. Scale bars represent 100 µm.

Prevalence of micro-adenomas and lack of advanced adenomas in the small intestines of the *AhCreER^T+^Apc^fl/fl^Brg^fl/fl^* mice as late as 100 days post induction indicated that this genetic environment favoured the development of lesions with a limited growth potential. Barker *et al.* [28] suggested that adenomas originating from intestinal stem cells had a higher tumourigenic potential compared to those derived from transit amplifying cells. To test whether stem cell-specific Brg1 loss could attenuate Wnt-driven adenoma formation we intercrossed mice expressing the *GFP-IRES-CreER^T2^* knock-in allele under control of the *Lgr5* promoter (further abbreviated as *Lgr5-GFP-CreER^T2^*) [23] with animals bearing targeted *Apc* and *Brg1* alleles. *Lgr5-GFP-CreER^T2+^Apc^fl/fl^* and *Lgr5-GFP-CreER^T2+^Apc^fl/fl^Brg^fl/fl^* mice were induced with four daily intraperitoneal injections of tamoxifen (initial dose of 3 mg was followed by three doses of 2 mg). Animals were aged for 420 days after induction or until they developed signs of ill health. Survival analysis of the cohorts revealed a substantial increase in survival of double knock-out mice compared to their *Lgr5-GFP-CreER^T2+^Apc^fl/fl^* counterparts (median survival 344 and 110 days post induction respectively, Figure 4A, Log-rank p<0.001, n = 14 for each cohort).

Immunohistochemical analysis of β-catenin expression in the small intestine of both *Lgr5-GFP-CreER^T2+^Apc^fl/fl^* and *Lgr5-GFP-CreER^T2+^Apc^fl/fl^Brg^fl/fl^* mice revealed a mixture of micro and macro-adenomas with nuclear β-catenin at a range of time points (Figure 4B). Immunohistochemical analysis of GFP expression in small intestinal tumours detected GFP positive cells in both macro and micro adenomas, implying activity of the *Lgr5-GFP-CreER^T2^* transgene and stem cell origin of both types of lesions (Figure 4B). This observation suggested that not all stem cell-derived adenomas were able to progress to advanced stages. Equally, we observed numerous micro adenomas devoid of GFP expression (not shown). These lesions were likely to arise from early progenitor cells, which might have lost self renewal ability, but retained some Cre-ER^T2^ recombinase expression.

Similar to the pattern observed in the small intestine of *AhCreER^T+^Apc^fl/fl^Brg^fl/fl^* mice, adenomas in the small intestine of *Lgr5-GFP-CreER^T2+^Apc^fl/fl^Brg^fl/fl^* mice retained Brg1 expression (Figure 4B) providing further support to the notion that Brg1 loss is incompatible with Wnt-driven adenoma formation.

## Discussion

### Brg1 acts as a positive regulator of Wnt signalling and is required for Wnt-driven adenoma formation in the small intestinal epithelium

We have demonstrated that inactivation of Brg1 resulted in reduced expression of Wnt target genes following activation of the canonical Wnt pathway via Apc deletion in the small intestinal epithelium. A number of studies have previously suggested that Brg1 facilitates trans-activation of Wnt target genes by activated β-catenin in cancer cell lines [12], [13], zebrafish [31] and during mammalian vascular development [32]. Our study provides the first evidence of the functional interaction between Brg1 and the Wnt pathway in the context of intestinal tumourigenesis using an *in vivo* system. Using transcriptome analysis, we identified sets of Wnt target genes which displayed differing levels of dependency on Brg1 function. Overall, we identified 548 genes that were deregulated upon Apc deletion in the small intestinal epithelium. Of these, 87 genes were Brg1 independent and 461 displayed a variable degree of dependency on Brg1 function, indicating that deregulation of the majority (85%) of Wnt responsive genes in the small intestinal epithelium depended (to differing degrees) on the presence of functional Brg1. This observation is in line with a previous report, which found a similar proportion (68%) of Wnt targets to rely on Brg1 for their response to Wnt activation in the HEK293T cell line [33].

Consistent with the requirement for Brg1 in the maintenance of the small intestinal stem cell population [14], we observed attenuated expression in a range of genes associated with the small intestinal stem cells following Brg1 loss in the context of aberrantly activated Wnt signalling. These genes included functionally validated stem cell markers such as *Lgr5*, *Ascl2* and *Olfm4*, suggesting that Brg1 loss was able to impair the expansion of the ‘stem-like’ cell population characteristic of Wnt-driven intestinal tumourigenesis [27]–[29]. Furthermore, expression levels of *Olfm4* in the small intestine of double knock-out mice were lower compared to that in both wild type and Apc-deficient epithelium, strongly suggesting a role of Brg1 in regulation of Olfm4 expression. Olfm4 is a secreted anti-apoptotic factor, which has been reported to be over-expressed in a variety of tumours [34]. Depletion of Olfm4 in gastric cancer cells has been reported to suppress proliferation and sensitise cancer cells to apoptosis [35], [36]. Olfm4 thus constitutes a potentially attractive therapeutic target, especially so in view of its secreted nature, which makes it a feasible target for monoclonal antibody therapies.

In addition to suppression of Wnt target genes, Brg1 loss also attenuated the physiological manifestations of Wnt activation in the intestinal epithelium, most notably the increased cell proliferation and mislocalisation of Paneth cells. Both Paneth cell quantity and position were restored to normal levels in the double knock-out epithelium, suggesting that Brg1 deletion was able to preserve physiological levels of EphB/Ephrin signalling in the context of Apc deficiency [37]. Consistent with this notion, our transcriptome analysis detected up-regulation of EphB2 expression in Apc-deficient intestine, but not in double knock-out epithelium, when compared to control samples. A similar effect of Wnt signalling attenuation on the Paneth cell mislocalisation has been previously demonstrated following loss of Mbd2 in the Apc-deficient small intestine [38]. Given the proposed role for Paneth cells as the intestinal stem cell niche [20], this effect of Brg1 loss on Paneth cell mislocalisation could contribute to the attenuated stem cell expansion in double knock-out intestine.

In the view of extensive role of Wnt signalling in tumourigenesis [1], Brg1 mediated modulation of the Wnt pathway may have implications for the development of novel therapeutic approaches. Here we report that loss of *Brg1* in the context of *Apc* deletion improved animal survival by preventing the formation of double mutant adenomas. All adenomas observed in the small intestine of double knock-out mice retained *Brg1* expression indicative of their origin from cells that had lost Apc but had escaped *Brg1* deletion. This implies an absolute requirement of functional Brg1 for Wnt-mediated tumourigenesis in this tissue. A similar relationship between Brg1 inactivation and a loss of tumour suppressor Snf5 (Ini1) has been reported by Wang *et al.* [39]. Simultaneous inactivation of *Brg1* and *Snf5* under control of the T-cell lineage-specific Lck-Cre recombinase resulted in decreased tumour incidence and a longer disease onset. Similar to our observations, all the Snf5-deficient tumours, which developed in double-mutant animals, retained *Brg1* expression [39].

Barker *et al.* [28] suggested a particular role for intestinal stem cells in Wnt-driven tumour initiation with non-stem cells giving rise to adenomas with limited growth capacity, while stem cell gene signature in human primary colorectal cancers was found to be associated with more aggressive phenotype [40]. In a similar fashion, Alcantara Llaguno *et al.* [41] reported neural stem cells as the cell of origin for malignant gliomas. Furthermore, a recent study has demonstrated a substantial survival advantage of genetic targeting of glioblastoma cancer stem cells using a neural stem cell marker *Nestin* [42]. Consistent with these reports, and in line with negative impact of Brg1 loss on long-term small intestinal stem cell survival, we observed a markedly improved survival upon stem cell-specific *Brg1* deletion in the context of aberrant Wnt activation. Notably, the above study made use of stem cell-specific expression of the toxic thymidine kinase transgene to target the sub-population of cancer cells bearing normal stem cell markers [42]. In contrast to this, we report successful tumour suppression by targeting a gene required for the physiological small intestinal stem cell maintenance, a relationship, which to our knowledge has not been previously reported. It should be noted that mice with stem cell-specific Apc loss alone in the present report exhibited longer survival times compared to those in the study by Barker *et al.* [28], which could be attributed to increased levels of silencing of the *Lgr5-GFP-CreER^T2^* transgene in our mice. Tumours in the small intestine of animals bearing the *Lgr5-GFP-CreER^T2^* transgene and targeted *Apc* alleles were mainly detected in the distal third of the small intestine with fewer lesions in the proximal part and very few tumours in between. Given that biallelic Apc deletion would fall within ‘high pathological Wnt’ scenario described in Leedham et al. [43], this tumour distribution was consistent with the gradient of the Wnt signal and stem cell density in the murine intestine.

It should be noted that given the concurrency of Brg1 and Apc deletion, results presented in this report pertain to cancer prevention rather than therapy. Additionally, Brg1 loss-driven elimination of intestinal stem cells is likely to be a major contributing factor to attenuated tumour burden in double knock-out mice and may therefore obscure the effects of Brg1 deletion on non-stem tumour cells. Importantly, this toxicity of Brg1 loss in respect to the small intestinal stem cell homeostasis constitutes a potential serious caveat to the use of Brg1 as a therapeutic target. However our data from the previous report [14] as well as from *AhCreER^T+^Apc^fl/fl^Brg^fl/fl^* and *Lgr5-GFP-CreER^T2+^Apc^fl/fl^Brg^fl/fl^* models in the present study demonstrate that partial Brg1 loss is well tolerated by the small intestinal epithelium, which is gradually repopulated with wild type cells. At the same time, partial loss of Brg1 was sufficient to reduce the tumour burden, suggesting that a therapeutic window may exist that would allow targeting Brg1 in the intestinal polyps, while allowing repopulation of the normal intestinal epithelium. A strategy that would allow for the efficient Brg1 deletion in existing adenomas or the use of a Brg1 inhibitor would be required to further address the effects of Brg1 in non-stem cell portion of established Wnt-driven tumours, as well as the potential therapeutic window of targeting Brg1.

In summary, we demonstrate using mouse models of intestinal cancer that Brg1 is essential for Wnt-driven tumourigenesis in the murine small intestine with attenuation of Wnt target gene expression and elimination of transformed stem cells as two likely mechanisms. Brg1 therefore constitutes a potential therapeutic target in cancers with an aberrantly activated Wnt pathway. Combined with our earlier observation that Brg1 is essential for stem cell maintenance in the small intestinal epithelium under the physiological conditions, these data may serve as a proof of concept that targeting the somatic stem cell as a cancer initiating cell may provide a valuable therapeutic approach, especially in the context of predisposition to Wnt-driven carcinogenesis, such as in Familial Adenomatous Polyposis patients.

## Materials and Methods

### Experimental animals

All experiments were carried out in accordance with Animals (Scientific Procedures) Act 1986 under project licenses 30/2246 and 30/2737 issued by UK Home Office. The study was approved by the Cardiff University Research Ethics Committee. Mice were maintained on an outbred background and genotyped as described previously for targeted Apc allele [15], Cre-ERT and Cre-ERT2 transgenes [19], targeted Brg1 allele [16]. Detailed induction protocols and dissection procedures are described in Text S1.

### Histological procedures

Detailed description of protocols for tissue fixation, processing, immunohistochemistry, and quantitative analysis of tissue sections is available in Text S1.

### Statistical analysis of histological data

All statistical tests except survival and cumulative distribution analyses were carried out using R software [44]. Scoring data were tested for normality using Shapiro-Wilk test and for equal variance using Levene's test. Normally distributed data with equal variance were tested for difference between means using one-way ANOVA. Where appropriate, p values were adjusted for multiple testing using TukeyHSD function in R. In cases of unequal variance between groups the difference between those groups was tested using t-test not assuming equal variance. Unless otherwise specified, pooled standard deviation from one-way ANOVA was used to represent error bars. Positional data were analysed for differences in distribution with Kolmogorov-Smirnov Z-test in SPSS (version 16.0.2). Kaplan-Meier survival curve and Log-rank survival analysis were carried out using GraphPad Prism (version 6).

### RNA extraction, quantitative RT-PCR and microarray analysis

Detailed description of the protocols and statistical methods used for RNA extraction, qRT-PCR and transcriptome analysis is available in Text S1. Microarray analysis was carried out using beadarray [45] and limma [26] packages from Bioconductor project. Microarray data for the study are publicly available from the GEO repository (http://www.ncbi.nlm.nih.gov/geo) under the series record GSE46129.

### Protein extraction and western blot analysis

A detailed protocol for obtaining epithelial-enriched population of cells for protein extraction and western-blotting is provided in Text S1. Protein was extracted from the epithelium-enriched small intestinal samples, separated, transferred and probed as described previously [46]. The primary antibodies used: mouse anti-active β-catenin (1∶2000; Millipore) and mouse anti-β-actin (1∶5000; Sigma). Anti-mouse horseradish peroxidase conjugated secondary antibody (1∶3000; GE Healthcare) and ECL or ECL Plus reagents (Amersham Biosciences) were used to detect the signal according to the manufacturer's manual.

### *In vitro* organoid culture

Small intestinal crypts from *VillinCreER^T2−^* controls, *VillinCreER^T2+^Apc^fl/fl^* and *VillinCreER^T2+^Apc^fl/fl^Brg^fl/fl^* mice were isolated and cultured as described in Sato *et al.* [47] with minor adjustments. Detailed procedures are described in Text S1.

## Supporting Information

Figure S1 Brg1 loss attenuates Wnt-driven apoptosis and cell proliferation in the small intestinal epithelium. (A, B) Scoring of the apoptotic bodies (A) and BrdU positive cells 2 hours post labelling (B) showed significantly reduced apoptosis levels and BrdU incorporation in *VillinCreER^T+^Apc^fl/fl^Brg^fl/fl^* mice compared to *VillinCreER^T+^Apc^fl/fl^* animals. Graphs are represented as mean ± group-wise standard deviation. Difference between means was tested by means of t-test for samples with unequal variance and adjusted for multiple testing. (C) Analysis of cumulative frequency of BrdU positive cells at each cell position along crypt-villus axis 2 hours after labelling revealed significant expansion of BrdU positive cells in *VillinCreER^T+^Apc^fl/fl^Brg^fl/fl^* (green line) and *VillinCreER^T+^Apc^fl/fl^* (orange line) mice compared to *VillinCreER^T−^* controls. This expansion was less pronounced in epithelium of *VillinCreER^T+^Apc^fl/fl^Brg^fl/fl^* mice compared to *VillinCreER^T+^Apc^fl/fl^* animals. For all comparisons Kolmogorov-Smirnov test p<0.001, n = 4.
(TIF)

Figure S2 Brg1 deletion specifically reverses gene expression changes induced by Apc deletion. (A, D) Correlation analysis of changes in gene expression revealed a strong negative correlation in expression patterns of genes in Apc deficient and double knock-out epithelium. The same pattern was observed for genome-wide analysis (A) and when applied to the genes from intestinal stem cell signature (D). Biological correlation is distinguished from technical correlation using “genas” function from Limma Bioconductor package [26]. (B) Genes deregulated by Brg1 loss in the control epithelium comprised a small fraction of genes affected by Brg1 deletion in the context of Apc loss (5/99 genes). (C) A small set of 16 genes that were disrupted by Brg1 loss regardless of Apc deletion were largely represented by direct Brg1 targets and were also misexpressed following Brg1 loss in normal intestinal epithelium (11/16 genes).
(TIF)

Table S1 Genes differentially expressed between *VillinCre^+^Apc^fl/fl^* and *VillinCre^−^* small intestinal epithelium.
(XLS)

Table S2 Genes differentially expressed between *VillinCre^+^Apc^fl/fl^Brg^fl/fl^* and *VillinCre^+^Apc^fl/fl^* small intestinal epithelium.
(XLS)

Table S3 Genes differentially expressed between *VillinCre^+^Apc^fl/fl^Brg^fl/fl^* and *VillinCre^−^* small intestinal epithelium.
(XLS)

Table S4 Overlapping and exclusive differentially expressed genes between *VillinCre^+^Apc^fl/fl^* vs *VillinCre^−^* (APCvsCTR) and *VillinCre^+^Apc^fl/fl^* vs *VillinCre^+^Apc^fl/fl^Brg^fl/fl^* (DKOvsAPC) datasets. Colours correspond to the colours in venn diagram in Figure 2G.
(XLS)

Table S5 Overlapping and exclusive differentially expressed genes between *VillinCre^+^Apc^fl/fl^* vs *VillinCre^−^* (APCvsCTR) and *VillinCre^+^Apc^fl/fl^Brg^fl/fl^* vs *VillinCre^−^* (DKOvsCTR) datasets. Colours correspond to the colours in venn diagram in Figure 2G.
(XLSX)

Table S6 Overlapping and exclusive differentially expressed genes between *VillinCre^+^Apc^fl/fl^Brg^fl/fl^* vs *VillinCre^−^* (DKOvsCTR) and *VillinCre^+^Apc^fl/fl^* vs *VillinCre^+^Apc^fl/fl^Brg^fl/fl^* (DKOvsAPC) datasets. Colours correspond to the colours in venn diagram in Figure 2G.
(XLS)

Table S7 Wnt target gene sets with differing levels of Brg1 dependency. Colours correspond to the colours in venn diagram in Figure 2G.
(XLS)

Table S8 Genes differentially expressed between *VillinCre^+^Brg1^fl/fl^* and *VillinCre^−^* small intestinal epithelium.
(XLS)

Table S9 Overlap of Brg1 targets (*VillinCre^+^Brg1^fl/fl^* vs *VillinCre^−^*) and differentially expressed genes in various gene sets. Colours correspond to the colours in venn diagram in Figure 2G.
(XLS)

Table S10 Small intestinal stem cell specific gene signature from Muñoz et al., (2012). Genes not detected in our array are highlighted in red.
(XLS)

Table S11 Stem cell signature genes differentially expressed between *VillinCre^+^Apc^fl/fl^* and *VillinCre^−^* small intestinal epithelium.
(XLS)

Table S12 Stem cell signature genes differentially expressed between *VillinCre^+^Apc^fl/fl^Brg^fl/fl^* and *VillinCre^−^* small intestinal epithelium.
(XLS)

Table S13 Stem cell signature genes differentially expressed between *VillinCre^+^Apc^fl/fl^Brg^fl/fl^* and *VillinCre^+^Apc^fl/fl^* small intestinal epithelium.
(XLS)

Table S14 Primers used for qRT-PCR analysis.
(XLS)

Text S1 Extended materials and methods.
(DOC)
