# Supplementary figures and images for: Brg1 Loss Attenuates Aberrant Wnt-Signalling and Prevents Wnt-Dependent Tumourigenesis in the Murine Small Intestine
Source: PLoS Genet. 2014 Jul 10;10(7):e1004453. doi: 10.1371/journal.pgen.1004453 (PMC4091792; doi:10.1371/journal.pgen.1004453)

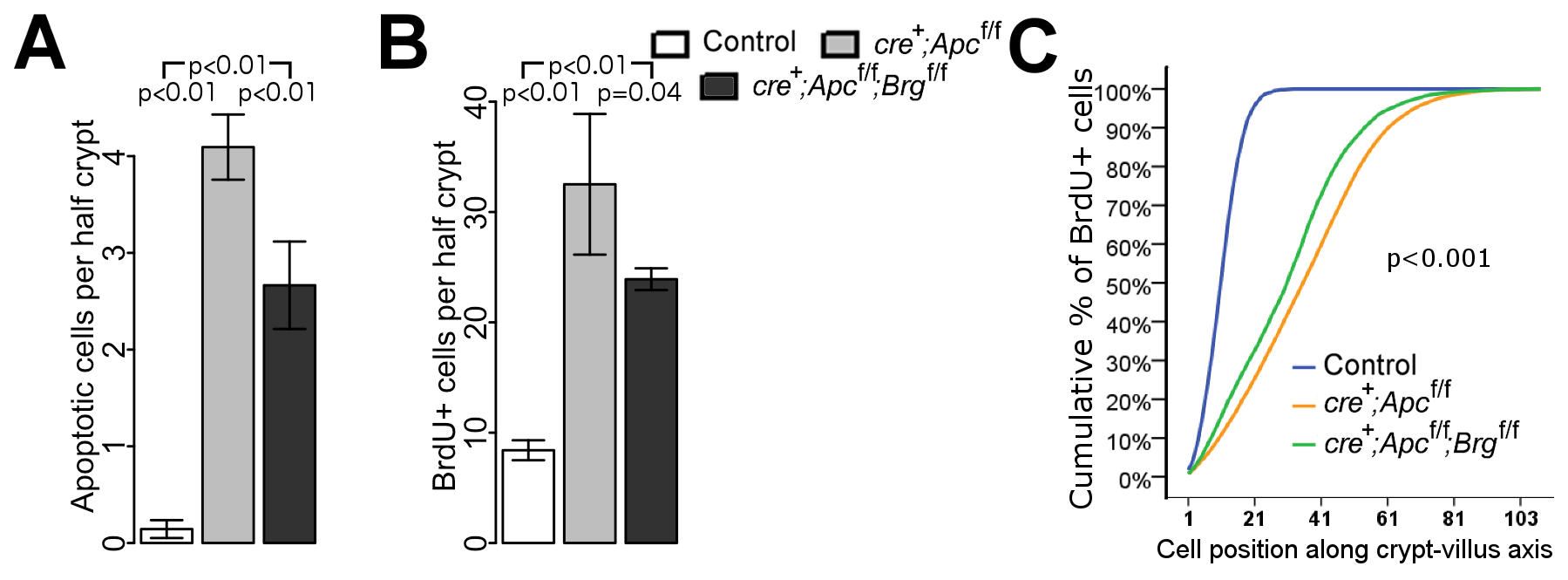

Supplement: Figure S1 — Brg1 loss attenuates Wnt-driven apoptosis and cell proliferation in the small intestinal epithelium. (A, B) Scoring of the apoptotic bodies (A) and BrdU positive cells 2 hours post labelling (B) showed significantly reduced apoptosis levels and BrdU incorporation in VillinCreERT+Apcfl/flBrgfl/fl mice compared to VillinCreERT+Apcfl/fl animals. Graphs are represented as mean ± group-wise standard deviation. Difference between means was tested by means of t-test for samples with unequal variance and adjusted for multiple testing. (C) Analysis of cumulative frequency of BrdU positive cells at each cell position along crypt-villus axis 2 hours after labelling revealed significant expansion of BrdU positive cells in VillinCreERT+Apcfl/flBrgfl/fl (green line) and VillinCreERT+Apcfl/fl (orange line) mice compared to VillinCreERT− controls. This expansion was less pronounced in epithelium of VillinCreERT+Apcfl/flBrgfl/fl mice compared to VillinCreERT+Apcfl/fl animals. For all comparisons Kolmogorov-Smirnov test p<0.001, n = 4. (TIF) [file pgen.1004453.s001.tif]

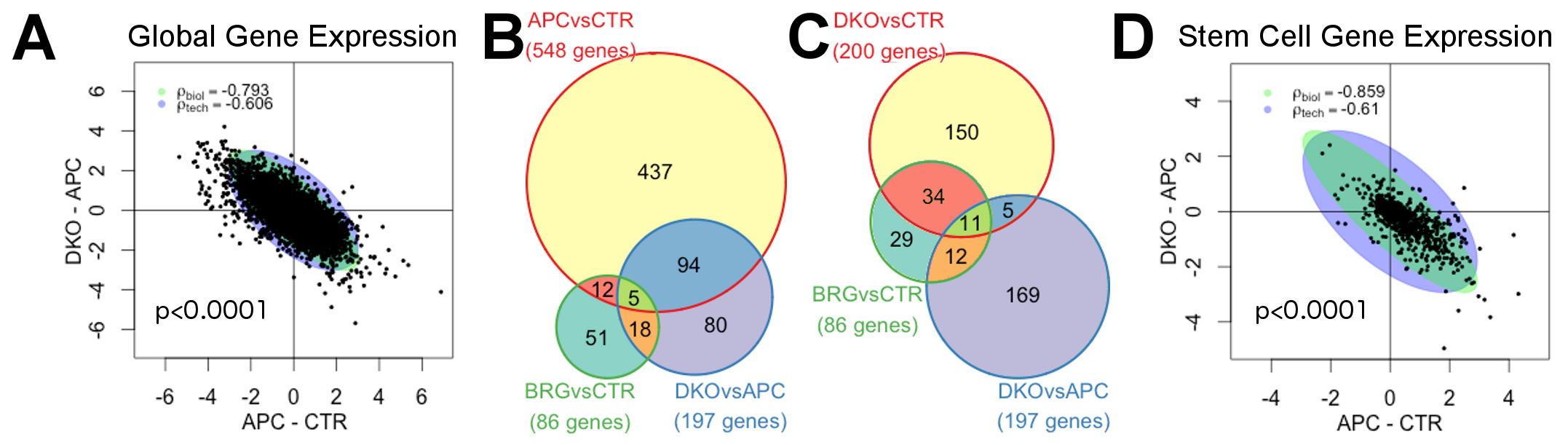

Supplement: Figure S2 — Brg1 deletion specifically reverses gene expression changes induced by Apc deletion. (A, D) Correlation analysis of changes in gene expression revealed a strong negative correlation in expression patterns of genes in Apc deficient and double knock-out epithelium. The same pattern was observed for genome-wide analysis (A) and when applied to the genes from intestinal stem cell signature (D). Biological correlation is distinguished from technical correlation using “genas” function from Limma Bioconductor package [26]. (B) Genes deregulated by Brg1 loss in the control epithelium comprised a small fraction of genes affected by Brg1 deletion in the context of Apc loss (5/99 genes). (C) A small set of 16 genes that were disrupted by Brg1 loss regardless of Apc deletion were largely represented by direct Brg1 targets and were also misexpressed following Brg1 loss in normal intestinal epithelium (11/16 genes). (TIF) [file pgen.1004453.s002.tif]
