# Supplementary material for: Brg1 Loss Attenuates Aberrant Wnt-Signalling and Prevents Wnt-Dependent Tumourigenesis in the Murine Small Intestine
Source: PLoS Genet. 2014 Jul 10;10(7):e1004453. doi: 10.1371/journal.pgen.1004453 (PMC4091792; doi:10.1371/journal.pgen.1004453)
Supplement: Text S1 — Extended materials and methods. (DOC) [file pgen.1004453.s017.doc]

**Supplemental Materials and Methods**

***Experimental animals***

Mice were maintained on an outbred background and genotyped as described previously for targeted *Apc* allele [15], *Cre* transgene [14], targeted *Brg1* allele [16]. Activity of *AhCreER^T^* recombinase [30] was induced by five bi-daily combined intraperitoneal injections of 80 mg/kg β-naphthoflavone and tamoxifen (Sigma). *VillinCreER^T^* recombinase [18] was induced by four daily injections of 80 mg/kg tamoxifen to achieve complete loss of the floxed alleles or a single 40 mg/kg injection to achieve partial loss of the targeted alleles. *Lgr5-GFP- CreER^T^* recombinase [23] was induced by a single injection of 3 mg followed by 3 daily injections of 2 mg of tamoxifen.

Upon dissection the whole gut was removed and flushed with 1X PBS. Second 8 cm of the small intestine corresponding to jejunum was cut into 2 cm pieces and bundled with surgical tape. Large intestine was cut open longitudinally, rolled and fixed with a hypodermic needle.

***Histology and immunohistochemistry***

Harvested tissues were fixed in ice-cold 10% neutral buffered formalin (Sigma) for no longer than 24 hours and embedded in paraffin following standard procedures. 5 μm sections were mounted on Poly-L-Lysine slides and stained with H&E for histological analysis or used for immunohistochemistry. Tissue sections were dewaxed and rehydrated by 5 min washes in 2 x Xylene, 2 x 100% ethanol, 1 x 95% ethanol and 1 x 70% ethanol. Antigen retrieval was carried out in citrate buffer (LabVision) in 99°C water bath for 20 min. Activity of endogenous peroxidases was blocked by incubating tissue sections for 5 min in peroxidase block solution (Envision+ Kit, DAKO). Slides were then blocked in appropriate normal serum (DAKO) in TBS + 0.1% Tween-20 (Cell Signalling Technology) for 30 min at room temperature and incubated overnight with a primary antibody in blocking solution. The following primary antibodies and dilutions were used: mouse anti-Brg1 (1:200; Santa Cruz), mouse anti-β-catenin (1:200; Transduction Laboratories), rabbit anti-Lysozyme (1:100; Neomarkers), rabbit anti-GFP (1:1000; AbCam), mouse anti-BrdU antibody (1:100; BD), rabbit anti-cleaved Caspase3 (1:200; Cell Signalling Technology), mouse anti-Ki67 (1:20; Vector Labs). Primary antibody binding was visualised using appropriate HRP-conjugated secondary antibody (1:200; DAKO) and 3.3’-diaminobenzidine according to manufacturer's instructions. Slides were routinely counterstained with Alcian Blue (1% (w/v) Alcian Blue (Sigma), in 3% (v/v) acetic acid (Fisher Scientific)) and Mayers Haemalum (R. A. Lamb) for 30 seconds each and dehydrated by reversing the dewaxing protocol. Coverslips were mounted over dehydrated sections using DPX mounting medium (R. A. Lamb)

***Scoring of crypt and villus length, apoptosis, mitosis, BrdU incorporation, and Lysozyme positive cells***

Apoptotic bodies, crypt and villus size were scored on H&E sections as described previously [19]. Cleaved Caspase 3, Ki67 and Lysozyme positive cells were scored on appropriately immunostained sections. For all the scoring, 50 half-crypts were analysed per mouse and at least 4 mice of each genotype were used. Positive cell positions were additionally recorded for Ki67 and Lysozyme analyses. Number of Cleaved Caspase 3 and Ki67 positive cells in the large intestinal lesions was normalised to the total number of cells in a lesion. For BrdU labelling experiment, mice were injected with 0.25 ml of BrdU (BD) 2 hours prior dissection. 50 half crypts per mouse were scored for BrdU labelled cells quantity and position on BrdU stained sections.

***RNA extraction, quantitative RT-PCR and microarray analysis***

Epithelium-enriched samples for mRNA extraction were obtained by scraping epithelium off the third 8 cm of the small intestine using surgical blade. Tissue was placed in 1 ml of Trizol (Invitrogen) and homogenised using Precellys24 homogeniser. Total mRNA was extracted using standard Trizol protocol, then purified and on-column DNAse treated using RNeasy mini kit (Qiagen). cDNA was synthesised using SuperScriptII (Invitrogen) and random primers (Promega) as described in http://www.untergasser.de/lab/protocols/cdna_synthesis_superscript_ii_v1_0.htm. We used DyNAmo HS SYBR Green qPCR kit (Finnzymes) to assess expression levels of *β-catenin, c-Myc, CD44, CyclinD1* and *Axin2* and TaqMan assay (custom designed by Applied Biosystems) for *Ascl2* and *Lgr5* following manufacturer's protocol. *β-actin* was used as a reference gene in all analysis. Reactions were run on StepOnePlus real-time PCR system (Applied Biosystems). Data were analysed using 2^ΔΔCt^ method (Livak and Schmittgen, 2001). Difference between means was tested using Mann-Whitney U test on ΔΔCt values.

**Primers used for qRT-PCR analysis**

| **Gene** | **Forward Primer** | **Reverse Primer** |
| --- | --- | --- |
| **b-catenin** | AGTCCTTTATGAATGGGAGCAA | TCTGAGCCCTAGTCATTGCATA |
| **b-actin** | TGTTACCAACTGGGACGACA | GGGGTGTTGAAGGTCTCAAA |
| **CD44** | ATCGCGGTCAATAGTAGGAGAA | AAATGCACCATTTCCTGAGACT |
| **c-myc** | CTAGTGCTGCATGAGGAGACAC | GTAGTTGTGCTGGTGAGTGGAG |
| **Cyclin D1** | ACGATTTCATCGAACACTTCCT | GGTCACACTTGATGACTCTGGA |
| **Axin2** | GCAGCTCAGCAAAAAGGGAAAT | TACATGGGGAGCACTGTCTCGT |

For transcriptome analysis total mRNA samples (n=4 for all genotypes except *VillinCreER^T+^Apc^fl/fl^*, where n=3) were labelled and hybridised to Mouse Ref8 v2 Illumina array by Central Biotechnology Services, Cardiff University. Expression analysis was carried out using a range of packages from Bioconductor project (Gentleman *et al*., 2004). Illumina array intensities data were extracted and adjusted for spatial artefacts using BASH algorithm in beadarray package [45]. Expression data were normalised using variance stabilising data transformation (vsn) method (Huber *et al*., 2002)). Differential expression analysis was carried out using limma package [26] and adjusted p values were obtained by Holm method (Holm, 1979). Differentially expressed probes were defined as those with adjusted p value <0.05. Gene names were assigned to Illumina probes using illuminaMousev2.db annotation package (Dunning *et al*.). Heatmaps and cluster analysis were performed as described in Gentleman *et al*. (2004). Venn diagram was built using R package Vennerable (version 2.2/r79). Stem cell signature gene set was obtained from Supplemental Materials in [24]. Stem cell specific gene enrichment was tested for each of the comparisons by roast function from limma package (Wu et al., 2010). Individual stem cell genes differentially expressed between the cohorts were obtained by overlapping the lists of differentially expressed genes with the genes in the stem cell signature. Correlation analysis of gene expression changes was carried out using “logFC” method in “genas” (Genuine Association of Gene Expression Profiles) function from limma package (Majewski et al., 2010) in order to distinguish between biological and technical correlation.

***Protein extraction and western blot analysis***

We obtained epithelial-enriched population of cells using Weiser solution extraction protocol. Briefly, the first 15 cm of the small intestine from the stomach was flushed with ice cold PBS, split longitudinally and placed in 15mls of a modified Weiser chelating solution (5.56mM disodium hydrogen orthophosphate, 8mM potassium dihydrogen orthophosphate, 96mM sodium chloride, 1.5mM potassium chloride, 27mM tri-Sodium citrate, 0.5mM dithiothreitol (DTT), 1.5% sucrose, 1% D-sorbitol, 6.07mM EDTA, 4.0mM EGTA, pH 7.3). Extraction was carried out as described in (Flint *et al*., 1991). Isolates were washed 3 times in ice cold PBS, snap frozen before being subjected to protein extraction.

***Intestinal in vitro organoid culture***

First 25 cm of the small intestine roughly comprising duodenum and jejunum were isolated, flushed with ice cold HBSS (Invitrogen) and cut longitudinally. Villi were gently scraped off using a cover slip. Intestine was then cut in 5 mm pieced, washed in ice cold HBSS and incubated in HBSS with 1:50 penicillin/streptomycin (Invitrogen) and 1:500 gentamycin (Sigma) for 15 min at room temperature. Intestine pieces were washed in HBSS, incubated in 8 mM EDTA (Sigma) in HBSS at room temperature for 5 min and at 4ºC for 20 min on a rotating platform. HBSS was replaced with advanced DMEM/F12 media (ADF) containing 1X Glutamax (Invitrogen) and 1:100 penicillin/streptomycin and pieces were shaken vigorously. Supernatant was then transferred into a clean tube and shaking was repeated twice or until supernatant became clear. Crypts were centrifuged at 700 rpm for 5 min, resuspended in ADF, passed through 70 μm cell strainer (BD) and centrifuged at 800 rpm for 3 min. Centrifugation at 800 rpm was repeated twice or until supernatant remained clear.

Crypts were resuspended in ADF and counted on low magnification. Crypts were plated in 50 μl of growth factor reduced Matrigel (BD) at 200-300 crypts per well of 24-well plate. Matrigel was overlaid with crypt culture media (ADF, 10 mM HEPES (Invitrogen), N-2 (Invitrogen), B27 without retinoic acid (Invitrogen), 50 ng/ml EGF (Invitrogen), and 100 ng/ml Noggin (Peprotech)) and media was changed every other day. Note that media used for culture of control organoids, but not Apc deficient of double knock-out organoids was supplemented with 1 μg/ml R-Spondin 1 (Sigma).

**References for Supplemental methods**

Dunning M., Lynch A. and Eldridge M.. illuminaMousev2.db: Illumina MouseWG6v2 annotation data (chip illuminaMousev2). R package version 1.12.1.

Flint N, Cove FL, Evans GS. 1991. A low-temperature method for the isolation of small-intestinal epithelium along the crypt-villus axis. Biochem J 280: 331–334.

Gentleman RC, Carey VJ, Bates DM, Bolstad B, Dettling M, Dudoit S, Ellis B, Gautier L, Ge Y, Gentry J, et al. 2004. Bioconductor: open software development for computational biology and bioinformatics. Genome Biol 5: R80.

Holm S. 1979. A Simple Sequentially Rejective Multiple Test Procedure. Scandinavian Journal of Statistics 6: 65–70.

Huber W, Von Heydebreck A, Sultmann H, Poustka A, Vingron M. 2002. Variance stabilization applied to microarray data calibration and to the quantification of differential expression. Bioinformatics 18: S96–S104.

Livak KJ, Schmittgen TD. 2001. Analysis of Relative Gene Expression Data Using Real-Time Quantitative PCR and the 2-[Delta][Delta]CT Method. Methods 25: 402–408.

Majewski IJ, Ritchie ME, Phipson B, Corbin J, Pakusch M, Ebert A, et al. Opposing roles of polycomb repressive complexes in hematopoietic stem and progenitor cells. Blood. 2010;116:731–9.

Muñoz J, Stange DE, Schepers AG, *et al.* The Lgr5 intestinal stem cell signature: robust expression of proposed quiescent ‘+4’ cell markers. *EMBO J* 2012;**31**:3079–91.

Wu D, Lim E, Vaillant F, et al. ROAST: rotation gene set tests for complex microarray experiments. Bioinformatics 2010;26:2176–82.
